# Genetic Variations in the Regulator of G-Protein Signaling Genes Are Associated with Survival in Late-Stage Non-Small Cell Lung Cancer

**DOI:** 10.1371/journal.pone.0021120

**Published:** 2011-06-17

**Authors:** Jingyao Dai, Jian Gu, Charles Lu, Jie Lin, David Stewart, David Chang, Jack A. Roth, Xifeng Wu

**Affiliations:** 1 Department of Epidemiology, The University of Texas MD Anderson Cancer Center, Houston, Texas, United States of America; 2 Department of Thoracic/Head and Neck Medical Oncology, The University of Texas MD Anderson Cancer Center, Houston, Texas, United States of America; 3 Department of Thoracic and Cardiovascular Surgery, The University of Texas MD Anderson Cancer Center, Houston, Texas, United States of America; University of Pennsylvania, United States of America

## Abstract

The regulator of G-protein signaling (RGS) pathway plays an important role in signaling transduction, cellular activities, and carcinogenesis. We hypothesized that genetic variations in RGS gene family may be associated with the response of late-stage non-small cell lung cancer (NSCLC) patients to chemotherapy or chemoradiotherapy. We selected 95 tagging single nucleotide polymorphisms (SNPs) in 17 RGS genes and genotyped them in 598 late-stage NSCLC patients. Thirteen SNPs were significantly associated with overall survival. Among them, rs2749786 of *RGS12* was most significant. Stratified analysis by chemotherapy or chemoradiation further identified SNPs that were associated with overall survival in subgroups. Rs2816312 of *RGS1* and rs6689169 of *RGS7* were most significant in chemotherapy group and chemoradiotherapy group, respectively. A significant cumulative effect was observed when these SNPs were combined. Survival tree analyses identified potential interactions between rs944343, rs2816312, and rs1122794 in affecting survival time in patients treated with chemotherapy, while the genotype of rs6429264 affected survival in chemoradiation-treated patients. To our knowledge, this is the first study to reveal the importance of RGS gene family in the survival of late-stage NSCLC patients.

## Introduction

Non-small cell lung cancer (NSCLC) is the leading cause of cancer mortality worldwide [1]. Over 45% of NSCLC patients present with unresectable late-stage (stage IIIA/B or stage IV) disease in the United States [2]. A combined modality therapy is the current standard of care for patients with stage III NSCLC with good performance status (performance score 0 or 1). Numerous clinical trials have shown that concurrent chemoradiation offers a significant survival advantage over sequential chemoradiation [3]. Although concurrent chemoradiotherapy significantly improves the survival of patients with locally advanced disease, the majority of patients still die within 5 years because of locoregional or distant disease progression [4]. The stage IV patients are usually offered palliative chemotherapy and supportive care [5]. There is a wide variability in patients' response to chemoradiation and clinicopathological variables alone do not provide satisfactory guidance for the decision of treatment strategy. The application of pharmacogenomics may improve the prediction of response and help clinicians determine cancer treatments for individual NSCLC patient according to his unique genetic background. Therefore, in this study, we aimed to identify genetic predictors for clinical outcomes of late stage NSCLC patients.

G proteins (guanine nucleotide-binding proteins) are important cellular signal transduction molecules that are expressed in all human cells [6], [7]. They are activated by G protein-coupled receptors (GPCRs) and thereby may transduce extracellular signals into the interior of a cell [8]. GPCRs are a family of seven-transmembrane domain receptors. When GPCRs traduce a signal inside the cell, the extracellular domain of GPCR first binds to the signal molecules, and then the intracellular domain of GPCR activates a heterotrimeric G-protein. The heterotrimeric G protein functions as “molecular switches” and can activate a cascade of signaling factors and downstream target activation [7]. This G protein-coupled biological process requires fine-tuning through accessory molecules such as the regulator of G-protein signaling (RGS) [9]. RGS proteins are a big family of over 30 intracellular proteins [10], which can negatively modulate GPCRs signaling pathways [11], [12]. RGS are multi-functional, GTPase-accelerating proteins that promote GTP hydrolysis by the alpha subunit of heterotrimeric G proteins, thereby inactivating the G protein and rapidly switching off GPCR signaling pathways[11]. All RGS proteins contain a RGS domain (also referred as “RGS-box”) ,which is required for their activities [13], and these RGS domains mediate the interaction with other signaling proteins, allowing RGS proteins to serve as signaling scaffolds [8]. Malfunctions of RGS proteins have been reported to be related to the pathogenesis of many common human diseases and drug addiction [14], [15], [16], [17]. Multiple RGS proteins were found differentially expressed in a variety of solid and hematological malignancies[18], [19], [20], [21], [22], [23], [24], [25], [26], [27], [28], [29], [30], [31], [32], [33], [34], [35], [36].

The single nucleotide polymorphisms (SNPs) of RGS have been associated with several human diseases, suggesting that genetic variation in the RGS pathway may play a significant role in these diseases' pathogenesis [37], [38]. Recently, RGS SNPs have also been reported to play important roles in lung cancer. For instance, SNPs in *RGS17* on chromosome 6q23-25 was associated with familial lung cancer susceptibility [39]. SNPs in *RGS2* and *RGS6* may modulate the risks of bladder and lung cancers [37], [40]. Whether genetic variants in the RGS pathway could influence clinical outcomes in patients with NSCLC remains unknown. In this study, we tested the hypothesis that genetic variations of RGS are associated with the survival of late-stage NSCLC patients receiving chemotherapy or chemoradiation.

## Results

We included 598 NSCLC patients in this study, with a mean age of 59.7 years (**Table 1**). Of the 598 patients, 456 were dead and 142 were alive. We found no significant difference in age (*P* = 0.884), ethnicity (*P* = 0.937), smoking status (*P* = 0.860), and pack-years of smoking (*P* = 0.926) between the two groups of patients (**Table 1**). However, we observed a significant difference in mortality status by gender (*P* = 0.002), clinical stage (*P* = 0.004), and performance status (*P* = 0.002) (**Table 1**).

**Table 1:** Distribution of demographic and clinical variables by survival status.

Variable	Dead (n = 456)	Alive (n = 142)	*P* value*
	Number (%)	Number (%)	
**Gender**			
Men	262 (81.11)	61 (18.89)	
Women	194 (70.55)	81 (29.45)	**0.002**
**Ethnicity**			
Caucasian	358 (76.17)	112 (23.83)	
African American	72 (75.79)	23 (24.21)	
Other	26 (78.79)	7 (21.21)	0.937
**Smoking status**			
Never smoker	84 (75.00)	28 (25.00)	
Former smoker	184 (74.72)	59 (24.28)	
Current smoker	188 (77.37)	55 (22.63)	0.860
**Clinical stage**			
IIIA	57 (69.51)	25 (30.49)	
IIIB (Dry)	100 (70.42)	42 (29.58)	
IIIB (Wet)	25 (60.10)	14 (35.90)	
IV	274 (81.79)	61 (18.21)	**0.004**
**Performance status**			
0	96 (67.13)	47 (32.87)	
1	254 (76.05)	80 (23.95)	
2-4	66 (89.19)	8 (10.81)	**<0.002**
**Therapy**			
Chemotherapy	295 (64.69)	60 (16.90)	
Chemoradiotherapy	104 (76.47)	32 (23.53)	
Both	57 (53.27)	50 (46.73)	**<0.001**
**Age, years (mean**±**SD)**	59.7±10.6	59.6±10.0	0.884
**Pack-years (mean**±**SD)**	36.6±30.4	36.9±31.3	0.926

*Significant *P* values are in bold font.

### Associations between SNPs and overall survival in late-stage NSCLC patients

A total of 13 SNPs in 6 genes were significantly associated with the risk of death at *P*<0.05 (**Table 2**). Among them, the variant alleles of four SNPs, rs7549021 and rs1056515 of *RGS5*, rs944343 of *RGS3*, and rs2749786 of *RGS12*, were associated with decreased risks of death, with adjusted HRs of 0.42 (95% CI, 0.22 to 0.83), 0.72 (95% CI, 0.54 to 0.97), 0.80 (95% CI, 0.67 to 0.95), and 0.58 (95% CI, 0.40 to 0.85), respectively. Other SNPs conferred increased risks of death. All SNPs in the *RGS1* gene were in linkage disequilibrium (with r^2^>0.8) with similar HRs in a dominant model.

**Table 2:** Significant SNPs associated with overall survival.

Gene and SNPs	No. of Dead/Alive	HR* (95% CI)	Smallest *P*	MST	Log-rank *P* value	No. of times in bootstrap samples *P*<0.05
*RGS3*						
rs944343&						99
GG	274/77	Reference		11.68		
CG	158/60	0.78 (0.63–0.97)	0.022435	14.21		
CCTrend	24/5	0.69 (0.43–1.10)0.80 (0.67–0.95)	0.118902*P_trend_* = 0.011796	18.29	0.1278	
*RGS4*						
rs6678136&						90
GG/GA	363/120	Reference		13.45		
AA	93/22	1.36 (1.07– 1.73)	0.012744	10.92	0.0364	
*RGS5*						
rs7549021&						93
AA/AG	444/132	Reference		12.83		
GG	12/10	0.42 (0.22–0.83)	0.012568	24.51	0.0647	
rs3820487&						95
CC/CA	431/141	Reference		13.45		
AA	25/1	1.81 (1.09–3.01)	0.022834	9.57	0.0001	
rs1056515						28
CC/CA	395/119	Reference		12.86		
AA	61/23	0.72 (0.54–0.97)	0.027911	13.62	0.1242	
*RGS12*						
rs2749786&						100
AA/AG	425/125	Reference		12.70		
GG	31/17	0.58 (0.40–0.85)	0.005532	26.84	0.0015	
*RGS22*						
rs2453627						41
CC	133/52	Reference		15.43		
CG/GG	323/90	1.26 (1.01–1.56)	0.039106	12.04	0.0247	
*RGS1*						
rs2760535#						47
GG	348/111	Reference		13.59		
GA/AA	108/31	1.31 (1.02–1.67)	0.031854	10.72	0.1558	
rs1323291#						11
AA	347/110	Reference		13.59		
AC/CC	109/33	1.28 (1.00–1.65)	0.049051	10.72	0.2181	
rs16834456#						47
CC	348/111	Reference		13.59		
CA/AA	108/31	1.31 (1.02–1.67)	0.031854	10.72	0.1558	
rs9427560#						47
AA	348/111	Reference		13.59		
AG/GG	108/31	1.31 (1.02–1.67)	0.031854	10.72	0.1558	
rs2816310#						11
CC	347/110	Reference		13.59		
CA/AA	109/33	1.28 (1.00–1.65)	0.049051	10.72	0.2181	
rs2816311#						11
AA	347/110	Reference		13.59		
AG/GG	109/33	1.28 (1.00–1.65)	0.049051	10.72	0.2181	

#Linkage SNPs.

Abbreviations: SNPs, single nucleotide polymorphism; No., number; HR, hazard ratio; CI, confidence interval;

MST, median survival time.

&SNPs which had bootstrap *P* values <0.05 at least 90% of times.

*HR adjusted by age, gender, ethnicity, smoking status and pack-years, performance status, clinical stage, and treatments.

The bootstrap re-sampling analysis was then performed for the 13 SNPs to internally validate the results. We found that only 5 SNPs, rs944343 (*RGS3*), rs6678136 (*RGS4*), rs7549021 (*RGS5*), rs3820487 (*RGS5*), and rs2749786 (*RGS12*), had bootstrap *P* values <0.05 at least 70 times out of 100 times (**Table 2**). The other SNPs had bootstrap *P* values <0.05 less than 50 times, indicating that those SNPs were likely false-positive results.

### Associations between SNPs and risk of death stratified by treatment

We then performed a stratified analysis by treatment modality, chemotherapy or chemoradiation (**Tables 3** **and** **4**). Nine SNPs were associated with overall survival in patients who received chemotherapy only, 5 of which had bootstrap *P* values <0.05 at least 70 times out of 100 times (**Table 3**). Among these five SNPs, three (rs2816312 [*RGS1*], rs10218752 [*RGS5*], and rs1122794 [*RGS11*]) were associated with an increased risk of death, with HRs of 1.80 (95% CI, 1.32 to 2.45), 1.76 (95% CI, 1.06 to 2.90), and 1.37 (95% CI, 1.07 to 1.77), respectively. On the other hand, rs944343 (*RGS3*) and rs1051013 (*RGS3*) were associated with a decreased risk of death, with HRs of 0.73 (95% CI, 0.57 to 0.94) and 0.77 (95% CI, 0.60 to 0.98), respectively. In Kaplan-Meier analyses, four of the five significant SNPs (rs2816312 [*RGS1*], rs944343 [*RGS3*], rs1051013 [*RGS3*], and rs1122794 [*RGS11*]) were significantly associated with altered median-survival time (MST) (log-rank *P* value <0.05) (**Table 3**).

**Table 3:** Stratified analysis by treatment modality: *Chemotherapy.*

SNPs	Gene	No. of Dead/Alive	HR* (95%CI)	Smallest *P*	MST	Log-rank *P* value	No. of times in Bootstrap samples P<0.05
rs2816312&	*RGS1*						100
AA		229/48	Reference		11.64		
AG/GG		66/12	1.80 (1.32–2.45)	0.000199	8.16	0.0134	
rs944343&	*RGS3*						99
GG		178/30	Reference		9.44		
GC/CC		117/30	0.73 (0.57–0.94)	0.013565	12.76	0.0009	
rs1051013&	*RGS3*						70
GG		186/33	Reference		9.57		
GA/AA		109/27	0.77 (0.60–0.98)	0.03534	12.70	0.0147	
rs10218752&	*RGS5*						97
AA/AG		278/59	Reference		10.92		
GG		17/1	1.76 (1.06–2.90)	0.027803	9.01	0.0861	
rs1122794&	*RGS11*						96
CC		193/48	Reference		11.55		
CA/AA		102/12	1.37 (1.07–1.77)	0.013747	9.47	0.0249	
rs12339493	*RGS3*						38
GG		239/43	Reference		10.53		
GA/AA		56/17	0.73 (0.53–0.99)	0.043020	12.40	0.0311	
rs7549021	*RGS5*						0
AA/AG		286/58	Reference		10.72		
GG		9/2	0.42(0.22–0.83)	0.013	9.57	0.6077	
rs2749786	*RGS12*						4
AA/AG		277/54	Reference		10.66		
GG		18/6	0.60 (0.37–1.00)	0.049813	11.71	0.015	
rs594149	*RGS16*						42
CC		205/35	Reference		10.59		
CG/GG		90/25	0.80 (0.61–1.04)	0.0984	11.71	0.0467	
*P* for trend				0.0446			
AA/AG		102/30	Reference		16.58		
GG		2/2	0.10 (0.01–0.78)	0.027524	24.51	0.0987	

Abbreviations: SNPs, single nucleotide polymorphism; No., number; HR, hazard ratio; CI, confidence interval; MST, median survival time.

& SNPs which had bootstrap *P* values <0.05 at least 70% of time.

*HR adjusted by age, gender, ethnicity, smoking status and pack-years, performance status, clinical stage.

# MST, median survival time (months)

**Table 4:** Stratified analysis by treatment modality: *Chemoradiation.*

SNPs	Gene	No. of Dead/Alive	HR* (95%CI)	Smallest *P*	MST	Log-rank *P* value	No. of times in Bootstrap samples P<0.05
rs2344673&	*RGS5*						88
GG		79/28	Reference		17.86		
GA/AA		25/4	1.86 (1.00–3.47)	0.049508	11.48	0.1024	
rs12127281&	*RGS5*						86
GG		59/25	Reference		19.14		
GA/AA		45/7	1.83 (1.09–3.10)	0.023387	12.30	0.0602	
rs12757054&	*RGS7*						73
GG		86/24	Reference		15.89		
GA/AA		18/8	0.48 (0.26–0.86)	0.013902	24.51	0.2311	
rs6429264&	*RGS7*						90
GG		82/28	Reference		19.28		
GA/AA		22/4	1.89 (1.06–3.38)	0.031160	12.37	0.0055	
rs6689169&	*RGS7*						96
AA		78/18	Reference		13.22		
AG/GG		26/14	0.47 (0.27–0.80)	0.005776	24.51	0.0441	
rs11586945	*RGS5*						3
GG		64/24	Reference		19.67		
GC/CC		40/8	1.63 (1.02–2.61)	0.040998	12.53	0.0623	
rs2999966	*RGS5*						0
CC/CA		96/27	Reference		16.05		
AA		8/5	0.38 (0.15–0.99)	0.047362	35.92	0.0642	
rs7549021	*RGS5*						0
AA/AG		102/30	Reference		16.58		
GG		2/2	0.10 (0.01–0.78)	0.027524	24.51	0.0987	

Abbreviations: SNPs, single nucleotide polymorphism; No., number; HR, hazard ratio; CI, confidence interval; MST, median survival time.

&SNPs which had bootstrap *P* values <0.05 at least 70% of time.

*HR adjusted by age, gender, ethnicity, smoking status and pack-years, performance status, clinical stage.

#MST, median survival time (months)

There were eight SNPs significantly associated with survival status in patients who received chemoradiation, five of which had bootstrap *P* values <0.05 more than 70 times out of 100 times (**Table 4**). Among these five SNPs, three (rs2344673 [*RGS5*], rs12127281 [*RGS5*], and rs6429264 [*RGS7*]) were associated with an increased risk of death, with HRs of 1.86 (95% CI, 1.00 to 3.47), 1.83 (95% CI, 1.09 to 3.10), and 1.89 (95% CI, 1.06 to 3.38), respectively; while rs12757054 (*RGS7*) and rs6689169 (*RGS7*) were associated with a decreased risk of death, with HRs of 0.48 (95% CI, 0.26 to 0.86) and 0.47 (95% CI, 0.27 to 0.80), respectively. Two of these 5 SNPs on *RGS7* (rs6429264 and rs6689169) were associated with altered MST for NSCLC patients (log-rank *P* = 0.0055 and 0.0441, respectively) (**Table 4**).

### Cumulative effects of the unfavorable genotypes on survival

**Figure 1:**
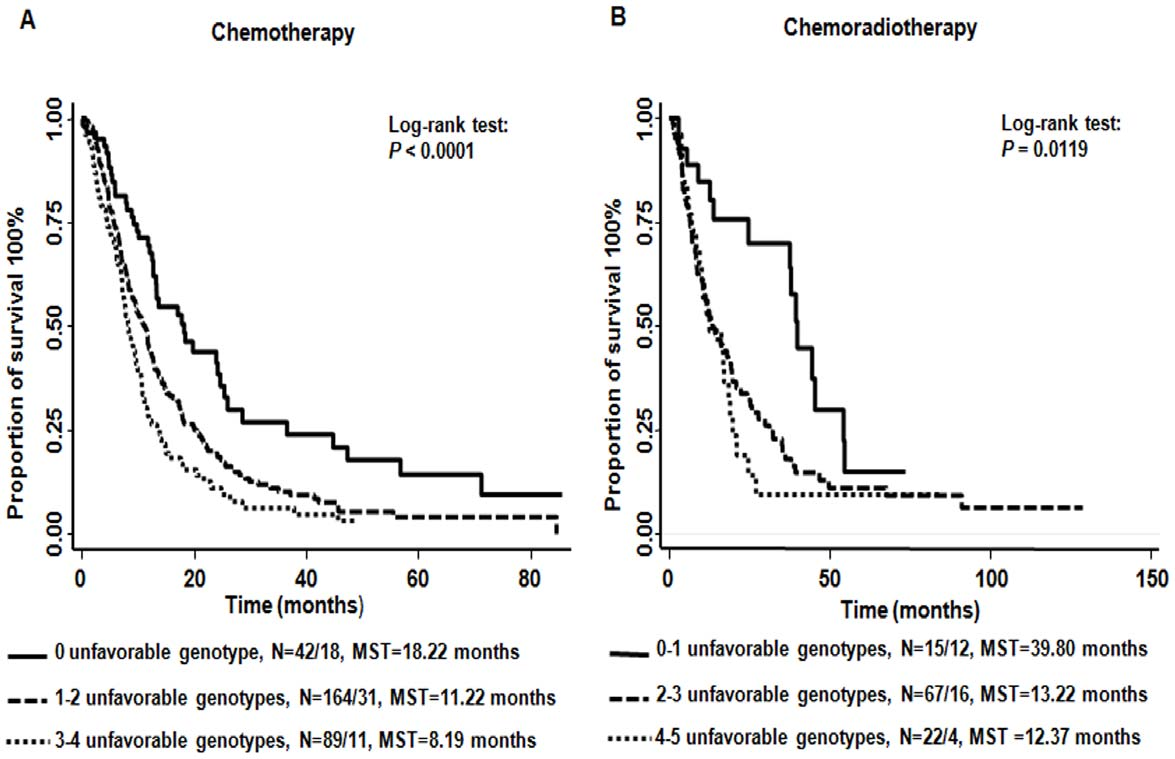
Kaplan-Meier estimates of the cumulative effect of unfavorable genotypes on NSCLC survival stratified by treatment. Solid line represents low-risk group, carrying 0 unfavorable genotypes in chemotherapy (**A**) and 0-1 unfavorable genotypes in chemoradiotherapy (**B**). Dashed line represents the medium-risk group, carrying 1-2 unfavorable genotypes in chemotherapy (**A**) and 2-3 unfavorable genotypes in chemoradiotherapy (**B**). Dotted line represents high risk group, carrying 3-4 unfavorable genotypes in chemotherapy (**A**) and 4–5 unfavorable genotypes in chemoradiotherapy (**B**). N: Number of Dead/Alive. MST: median survival time.

We further assessed the cumulative effects of the unfavorable genotypes in either treatment groups using the SNPs with bootstrap *P* values <0.05 at least 70 times out of 100 times in each group (**Table 5**). There were significant gene-dose effects in patients receiving both treatments (**Table 5**). In those patients receiving chemotherapy only and taking the low-risk reference group as reference (0 unfavorable genotypes), the medium-risk (1 or 2 unfavorable genotypes) and the high-risk groups (3 or 4 unfavorable genotypes) had 1.69-fold (95% CI, 1.19 to 2.41; *P* = 0.004) and 2.52-fold (95% CI, 1.71–3.71; *P*<0.001) increased risk of death, respectively (*P* for trend <0.001). The MST for the medium-risk and the high-risk groups were 11.22 months and 8.19 months, respectively, compared to 18.22 months for the low-risk reference group (log-rank *P* value <0.0001). Similar dose-response trends were observed among patients receiving chemoradiation (**Table 5** and **Figure 1**).

**Table 5:** The cumulative effects of unfavorable genotypes on survival.

Chemotherapy						Chemoradiation					
No. of unfavorable genotypes	No. dead/alive	HR* (95% CI)	*P* value	MST#	Log-rank *P* value	No. of unfavorable genotypes	No. dead/alive	HR* (95% CI)	*P* value	MST#	Log-rank *P* value
0	42/18	Reference		18.22		0–1	15/12	Reference		39.80	
1–2	164/31	1.69(1.19–2.41)	0.004	11.22		2–3	67/16	3.48(1.70–7.11)	0.001	13.22	
3–4	89/11	2.52(1.71–3.71)	<0.001	8.19	<0.0001	4–5	22/4	5.07(2.04–12.64)	<0.001	12.37	0.0119
			P_trend_<0.001						P_trend_<0.001		

*HR adjusted by age, gender, ethnicity, smoking status and pack-years, performance status, clinical stage.

#MST, median survival time (months)

### Higher-order gene-gene interactions

**Figure 2:**
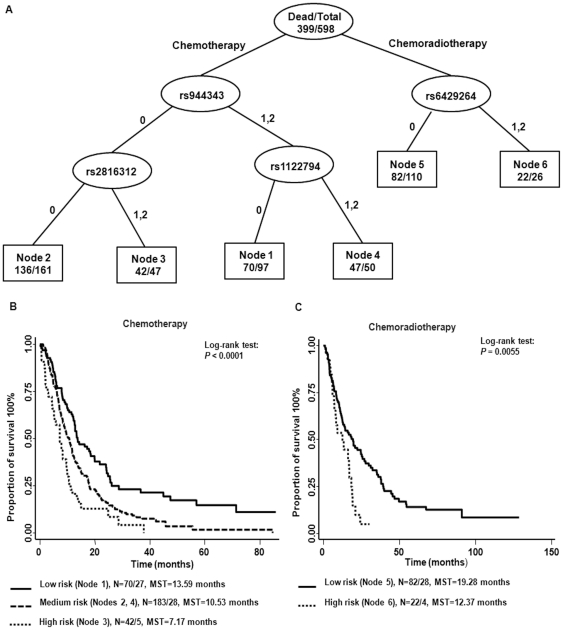
Survival tree analyses and Kaplan-Meier estimates of the significant RGS SNPs associated with survival. (**A**) Survival tree analyses; 0:homozygous wild type genotype; 1:heterozygous genotype; 2:homozygous variant genotype. (**B**) and (**C**) represent the survival curves of the risk group in chemotherapy group and chemoradiotherapy group; N: Number of Dead/Alive. MST: median survival time.

The results of STREE program analysis for the interaction of the 10 bootstrap-validated significant SNPs (the SNPs which had bootstrap P values<0.05 at least 70% of time in **Tables 3** **and** **4**) in stratified analysis were presented in Figure 2. Survival tree analysis resulted in four terminal nodes in the chemotherapy group and two terminal nodes in the chemoradiation group (**Figure 2A**). In the chemotherapy group, the initial split was rs944343 (*RGS3*), and subsequent splits were rs2816312 (*RGS1*) and rs1122794 (*RGS11*). Different nodes had different percentages of death event. To assess the risk associated with each of the terminal nodes, node 1 in the chemotherapy branch was taken as the reference group, composed of individuals with the heterozygous and the homozygous variant genotypes of rs944343 (*RGS3*) and the homozygous wild-type genotype of rs1122794 (*RGS11*). Compared with the reference group, patients in the terminal nodes in the chemotherapy group had HRs ranging from 1.49 to 2.92. Patients in node 1 had the longest MST of 13.58 months. The highest at-risk group (node 3), which was composed of patients carrying the homozygous wild-type genotype of rs944343 (*RGS3*) and variant-containing genotypes of rs2816312 (*RGS1*), had a HR of 2.92 (95% CI, 1.92 to 4.44). The MSTs were shown to be significantly different in Kaplan-Meier survival analysis (log-rank *P* value <0.0001) (**Figure 2B** and **Table 6**). In the chemoradiation group, there was only one additional split. Compared to the patients with the homozygous wild-type genotype of rs6429264 (*RGS7*) (node 5), who had an MST of 19.28 months, the patients carrying variant-containing genotypes of rs6429264 (*RGS7*) exhibited a 1.89-fold increased risk of death (95% CI, 1.06 to 3.38), with an MST of 12.37 months (**Figure 2C** and **Table 6**).

**Table 6:** Cox proportional hazards regression model in late-stage NSCLC patients based on the Survival tree analysis.

Risk Group	Dead (N%)	Alive (N%)	HR* (95%CI)	P-value	MST#	Log-rank P value
***Chemotherapy***						
Low (Node 1)	70 (72.16)	27 (27.84)	Reference		13.59	
Medium (Nodes 2, 4)	183 (86.73)	28 (13.27)	1.55 (1.16–2.09)	0.003	10.53	
High (Node3)	42 (89.36)	5 (10.64)	2.94 (1.93–4.47)	<0.001	7.17	<0.0001
***Chemoradiation***						
Low (Node 5)	82 (74.55)	28 (25.45)	Reference		19.28	
High (Node 6)	22 (84.62)	4 (15.38)	1.89 (1.06–3.38)	0.031	12.37	0.0055

*HR adjusted by age, gender, ethnicity, smoking status and pack-years, performance status, clinical stage.

#MST, median survival time (months)

## Discussion

In this study, we found that genetic variations in RGS genes were associated with overall survival in late-stage NSCLC patients. Our findings also reinforced the importance of evaluating the cumulative and interaction effects of genetic variations when predicting clinical outcomes of patients with NSCLC.

NSCLC patients are mostly treated with the platinum-based chemotherapy, often in combination with radiation therapy. The platinum-based chemotherapy may be related to several cellular pathways, such as the DNA damage/repair, cell cycle control, and apoptosis pathways [41]. However, there has been no study reporting that RGS is involved in the platinum-based chemotherapy related pathways.

NSCLC cells can invade adjacent tissues and metastasize to nonadjacent organs and tissues, processes that may be attributed to altered cellular signaling pathways [42], [43]. Oncogenic transformation is often the direct result of mutations of the signaling molecules, which constitute these pathways. In this study, 5 SNPs were associated with the overall risk of death with bootstrap *P* values <0.05 at least 90 times out of 100 times. Three of these 5 SNPs, rs6678136 (*RGS4*), rs3820487 (*RGS5*) and rs2749786 (*RGS12*) conferred significantly different MST in Kaplan-Meier curve (**Table 4**). Previous studies reported that *RGS4* gene expression were associated with invasion of several cancer [36], [44]. In addition, RGS4 protein acts as an inhibitor of epithelial and endothelial cell tubulogenesis by regulating mitogen-activated protein kinases and vascular endothelial growth factor signaling, thereby inhibiting cell proliferation, migration, and invasion [45]. Xiao *et a.l* reported that multiple SNPs in combination in *RGS5* may confer risk for hypertension in Chinese population [46]. *RGS5* was reported to be a key modulator of tumor pericyte maturation and play a pivotal role in tumor neovascularization [9]. *RGS5* knockout mice showed larger tumor burden and earlier death which may be caused by pericyte maturation and vascular normalization [33]. RGS5 has also been identified as a broadly expressed tumor antigen in multiple types of cancer [47]. RGS12 is a large RGS protein with multiple functional domains such as PDZ, PTB (phospho-tyrosine binding) and Rap binding domains [48]. PDZ domain of RGS12 interacts with a GPCR, CXCR2, and thereby contributes to the GAP action of RGS12 on CXCR2-mediated G-protein signals [49]. Therefore, it is biologically plausible that RGS4, RGS5, and RGS12 are associated with lung cancer survival. The functions of the significant SNPs on these genes are not clear since they are most likely tagging SNPs. Future studies are needed to find the causal SNPs.

In stratified analyses, 5 SNPs in the chemotherapy group and another 5 in the chemoradiation group were associated with the risk of death with bootstrap *P* values <0.05 at least 70 times out of 100 times. The genotypes of four SNPs: rs2816312 (*RGS1*), rs944343 (*RGS3*), rs1051013 (*RGS3*), and rs1122794 (*RGS11*) were found to be significantly associated with MST in chemotherapy group. The most significant one was rs944343 (Log-rank *P* = 0.0009), which was a tagging SNP located at the 3′ flanking region of *RGS3*. Increased RGS3 expression has been used as a diagnostic marker for soft tissue sarcoma and was associated with resistance to chemotherapy in breast cancer [50], [51]. In addition, RGS3 has been reported to modulate glioma cell mobility [36]. The other host genes of SNPs in chemotherapy group, *RGS1*, and *RGS11*, have also been associated with the etiology and prognosis of cancer. Rangel *et al*. reported that RGS1 may be a prognostic marker in melanoma progression and its expression was associated with survival for melanoma patients [20]. Martinez-Cardus, *et al*. reported that RGS11 expression was significantly associated with the resistance to platinum therapy in colorectal cancer [52]. These studies support the role of RGS1, RGS3, and RGS11 in lung cancer prognosis. There were only two SNPs in chemoradiation group, rs6429264 and rs6689169, significantly associated with MST (log-rank *P* = 0.0055 and 0.0441, respectively). both of which are tagging SNP and located in *RGS7*. Several studies demonstrated that tumor necrosis factor-α, a major inflammation cytokine that plays an important role in many human cancers, can rapidly activate the expression level of RGS7 [53], [54]. The mechanisms by which these genotypes determine their phenotypes and affect the outcome of NSCLC are not clear. Further studies are warranted to identify the causal variants and the biologic mechanisms underlying our observed associations.

We also observed cumulative effects of RGS SNPs on the survival of late-stage NSCLC patients. In addition, we used survival tree analysis to identify interactions among these SNPs. These gene-gene interactions resulted in four terminal nodes with different risks of death in the chemotherapy-only group and two terminal nodes with different risks of death the chemoradiation group. The cumulative-effects analysis and survival tree analysis may allow us to identify more powerful prognostic or predictive markers and signatures based on the combination of each patient's genetic variations. It should be noted that these types of analyses were exploratory, and the results need to be validated in independent studies.

There are a few strengths to our study. First, our current pathway-based approach is a logical extension of the candidate gene approach and avoids the requirement of much larger sample size by genome-wide association study. Second, we have collected a relative large population of NSCLC patients from the same institution. The uniform standard operation procedures in the cancer identification, pathological staging, and even strategy determination for cancer treatment made our findings more comprehensive and applicable to future clinical studies. Third, we have performed internal statistical validation by a bootstrap resampling procedure to minimize false discoveries. Fourth, we have performed exploratory gene-gene interaction analysis to establish a novel combination of SNPs to predict the outcome of NSCLC patients for their therapy, which could help clinicians in determining the optimal personalized treatment and the quality of care for survival.

To the best of our knowledge, this is the first study investigating the association of genetic variations in RGS family with survival for NSCLC. Our results have provided not only SNP-based analysis, but also a more comprehensive pathway-based approach in the clinical outcome prediction for NSCLC patients who underwent chemotherapy or chemoradiation. Future independent validation in larger population and detailed functional assays are necessary before these findings can be translated to the clinics.

## Methods

### Ethics Statement

All patients signed a written informed consent and this study has been reviewed and approved by the Institutional Review Board (IRB) of MD Anderson.

### Study population and collection of epidemiologic and clinical data

A total of 598 patients with late-stage NSCLC, including stages IIIA, IIIB (Dry), IIIB (Wet), and IV, recruited between 1995 and 2007 from an epidemiological lung cancer study being conducted at The University of Texas MD Anderson Cancer Center. None of them had been previously treated by surgery chemotherapy, and/or radiotherapy before enrollment into the study. All participants had completed a risk factor questionnaire that collected data on demographic characteristics, tobacco use history, occupational and environmental exposures, prior medical history, and any history of cancer in first-degree relatives, and also had donated a 40-ml blood sample for genotyping. We extracted the clinical information from the patients' medical records of their co-morbid conditions, tumor size, clinical stage, pathologic stage, histological type, tumor grade, treatment type, tumor recurrence, survival, and tumor progression for all the analyses. The median follow-up time was 11.8 months.

### SNP selection and genotyping

A comprehensive panel of cancer-related genes including RGS gene family was identified and classified in each specific pathway according to their major reported functions. In the gene list, seventeen genes in the RGS family (*RGS 1–5*, *7–14*, *16*, *18*, *20*, and *22*) were selected for this panel. The detailed procedure for compiling the panel of genes and SNPs were reported previously [55]. Genomic DNA was extracted from the peripheral blood lymphocytes of the patients' blood samples, and all the genotyping work were performed according to the standard protocol provided by Illumina Inc. Then the results of genotyping were automatically generated by the Illumina's BeadStudio software. Finally, 95 SNPs in the RGS pathway were selected and successfully genotyped in these patients, as shown in **Table S1** in the Supporting Information.

### Statistical analysis

STATA statistical software (StataCorp LP, College Station, TX) version 10.2 was used for the analysis of hazard ratios (HRs), *P* values, median survival time (MST), *P* values for log-rank test and Kaplan-Meier survival estimate. χ^2^ test (for categorical variables) and Student's *t*-test (for continuous variables) were used to assess differences in variables between dead and alive patients. For each SNP, the risk of death as a hazard ratio (HR) and 95% confidence interval (CI) were estimated with the Cox proportional hazards regression model. In addition, multivariate adjustment was used to control for potential confounding factors (age, gender, ethnicity, smoking status and pack-years, performance status, clinical stage, and treatment). For each SNP, the genetic distribution were assessed by three genetic models (dominant, recessive, and additive), and the model with the smallest *P* value was selected as the best-fitting model [56]. To validate the results, the bootstap resampling method was used. For each bootstrap sample drawn from the original data set, 100 bootstrap samples were generated. We obtained the *P* value for each SNP among the dominant, recessive, and additive models. The cumulative effects of different genotypes were calculated by summing up the individual effects of significant SNPs, that is, SNPs that showed significant association in single-SNP analysis and also had a bootstrap *P* value <0.05 at least 70 times. We used Cox proportional hazards regression model to estimate the HRs and 95% CIs. The Kaplan-Meier method and the log-rank test were used to estimate their effects on survival duration for these SNPs. Finally, the STREE program (http://masal.med.yale.edu/stree/) was used to perform survival tree analysis for the higher-order gene-gene interactions of the SNPs. For these analyses, we only included SNPs that had been validated internally by bootstrapping. A two-sided *P*<0.05 was considered statistically significant.

## Supporting Information

Table S1 **NPs in the RGS pathway.**
(DOC)
