# Supplementary material for: Genetic Variations in the Regulator of G-Protein Signaling Genes Are Associated with Survival in Late-Stage Non-Small Cell Lung Cancer
Source: PLoS One. 2011 Jun 17;6(6):e21120. doi: 10.1371/journal.pone.0021120 (PMC3117866; doi:10.1371/journal.pone.0021120)
Supplement: Table S1 — NPs in the RGS pathway. (DOC) [file pone.0021120.s001.doc]

**Table 1. SNPs in the RGS pathway**

| **SNP** | **Gene** | **Minor Allele** | **Model*** | **HR (95%CI)** | ***P*-value** |
| --- | --- | --- | --- | --- | --- |
| rs10218752 | *RGS5* | G | Rec | 1.52 (1.00-2.30) | 0.051574 |
| rs10489966 | *RGS16* | C | Rec | 0.79 (0.55-1.14) | 0.210647 |
| rs1051013 | *RGS3* | A | Dom | 0.83 (0.68-1.01) | 0.067441 |
| rs1056515 | *RGS5* | A | Rec | 0.72 (0.54-0.97) | 0.027911 |
| rs10752878 | *RGS16* | C | Dom | 1.02 (0.82-1.27) | 0.875662 |
| rs10753605 | *RGS5* | G | Dom | 1.07 (0.88-1.30) | 0.509275 |
| rs10759 | *RGS4* | A | Rec | 1.04 (0.72-1.50) | 0.82234 |
| rs10917682 | *RGS5* | G | Dom | 1.11 (0.81-1.53) | 0.511904 |
| rs10917690 | *RGS5* | G | Rec | 0.83 (0.58-1.17) | 0.279347 |
| rs10917691 | *RGS5* | A | Dom | 0.85 (0.68-1.06) | 0.142879 |
| rs10926466 | *RGS7* | G | Dom | 1.14 (0.93-1.39) | 0.220532 |
| rs11198978 | *RGS10* | A | Dom | 1.17 (0.85-1.61) | 0.343013 |
| rs11199005 | *RGS10* | A | Dom | 1.03 (0.80-1.34) | 0.800527 |
| rs1122794 | *RGS11* | A | Dom | 1.22 (0.99-1.51) | 0.058074 |
| rs1144566 | *RGS16* | A | Dom | 0.77 (0.52-1.13) | 0.176562 |
| rs11585883 | *RGS5* | G | Dom | 0.77 (0.57-1.05) | 0.103556 |
| rs11586945 | *RGS5* | C | Dom | 1.15 (0.92-1.44) | 0.206205 |
| rs11654243 | *RGS9* | A | Rec | 0.87 (0.60-1.27) | 0.466472 |
| rs11658673 | *RGS9* | G | Rec | 0.87 (0.60-1.27) | 0.466472 |
| rs11658773 | *RGS9* | G | Rec | 0.87 (0.60-1.27) | 0.466472 |
| rs12035879 | *RGS5* | A | Add | 0.90 (0.77-1.04) | 0.153535 |
| rs12038803 | *RGS7* | A | Dom | 1.20 (0.99-1.47) | 0.067224 |
| rs12087556 | *RGS4* | A | Dom | 1.17 (0.85-1.61) | 0.333513 |
| rs12127281 | *RGS5* | A | Add | 1.14 (0.97-1.35) | 0.108712 |
| rs12138022 | *RGS5* | G | Rec | 0.76 (0.54-1.07) | 0.118279 |
| rs12339493 | *RGS3* | A | Dom | 0.78 (0.60-1.01) | 0.064241 |
| rs12406291 | *RGS13* | C | Dom | 0.96 (0.71-1.31) | 0.813459 |
| rs12752933 | *RGS13* | G | Dom | 1.16 (0.93-1.45) | 0.197415 |
| rs12757054 | *RGS7* | A | Dom | 0.78 (0.58-1.05) | 0.105158 |
| rs1323291 | *RGS1* | C | Dom | 1.28 (1.00-1.65) | 0.049051 |
| rs1395960 | *RGS5* | A | Rec | 1.65 (0.98-2.79) | 0.060429 |
| rs1507735 | *RGS5* | A | Dom | 1.05 (0.86-1.28) | 0.619084 |
| rs1507755 | *RGS5* | G | Dom | 1.15 (0.86-1.54) | 0.346942 |
| rs1547624 | *RGS1* | A | Dom | 0.93 (0.76-1.14) | 0.499181 |
| rs16829458 | *RGS2* | A | Dom | 0.96 (0.73-1.26) | 0.763738 |
| rs16834456 | *RGS1* | A | Dom | 1.31 (1.02-1.67) | 0.031854 |
| rs16840577 | *RGS7* | A | Dom | 0.92 (0.73-1.15) | 0.45369 |
| rs16844152 | *RGS12* | A | Dom | 0.87 (0.64-1.19) | 0.388559 |
| rs16850474 | *RGS5* | G | Dom | 0.83 (0.61-1.14) | 0.249203 |
| rs16850625 | *RGS5* | A | Dom | 1.07 (0.86-1.32) | 0.555883 |
| rs16960920 | *RGS9* | A | Rec | 0.72 (0.45-1.13) | 0.153292 |
| (continued on following page) | | | | | |

**Supplementary Table 1. All-95 SNPs (Continued)**

| **SNP** | **Gene** | **Minor Allele** | **Model*** | **HR (95%CI)** | ***P*-value** |
| --- | --- | --- | --- | --- | --- |
| rs16960957 | *RGS9* | A | Rec | 0.87 (0.60-1.27) | *0.466472* |
| rs1819741 | *RGS2* | G | Rec | 1.20 (0.83-1.72) | *0.338317* |
| rs1890397 | *RGS2* | A | Add | 0.91 (0.80-1.05) | *0.200317* |
| rs1890398 | *RGS2* | A | Dom | 1.06 (0.87-1.29) | *0.583995* |
| rs1890399 | *RGS2* | T | Dom | 1.06 (0.87-1.29) | *0.554476* |
| rs2036702 | *RGS5* | A | Dom | 1.12 (0.90-1.38) | *0.30767* |
| rs2179653 | *RGS2* | A | Dom | 1.12 (0.90-1.40) | *0.299058* |
| rs2247482 | *RGS18* | G | Rec | 1.15 (0.85-1.54) | *0.364984* |
| rs2269497 | *RGS12* | G | Dom | 0.81 (0.54-1.21) | *0.30441* |
| rs2344673 | *RGS5* | A | Dom | 1.18 (0.93-1.49) | *0.169776* |
| rs2453627 | *RGS22* | G | Dom | 1.26 (1.01-1.56) | *0.039106* |
| rs2662774 | *RGS5* | A | Rec | 0.88 (0.57-1.36) | *0.557204* |
| rs2746071 | *RGS2* | G | Rec | 1.26 (0.90-1.77) | *0.172813* |
| rs2749786 | *RGS12* | G | Rec | 0.58 (0.40-0.85) | *0.005532* |
| rs2760535 | *RGS1* | A | Dom | 1.31 (1.02-1.67) | *0.031854* |
| rs2815287 | *RGS5* | C | Add | 1.14 (0.99-1.33) | *0.07436* |
| rs2816310 | *RGS1* | A | Dom | 1.28 (1.00-1.65) | *0.049051* |
| rs2816311 | *RGS1* | G | Dom | 1.28 (1.00-1.65) | *0.049051* |
| rs2816312 | *RGS1* | G | Dom | 1.28 (1.00-1.64) | *0.053834* |
| rs2841993 | *RGS5* | A | Rec | 1.52 (0.99-2.35) | *0.055672* |
| rs2940251 | *RGS4* | G | Rec | 1.06 (0.77-1.46) | *0.724613* |
| rs2940677 | *RGS5* | A | Rec | 0.90 (0.63-1.28) | *0.542968* |
| rs2984920 | *RGS1* | A | Dom | 0.85 (0.68-1.06) | *0.159666* |
| rs2999966 | *RGS5* | A | Rec | 0.74 (0.54-1.02) | *0.064836* |
| rs3735976 | *RGS22* | G | Dom | 0.87 (0.68-1.10) | *0.244795* |
| rs3747813 | *RGS3* | A | Dom | 0.87 (0.59-1.26) | *0.45882* |
| rs3795617 | *RGS13* | A | Rec | 0.88 (0.69-1.12) | *0.293827* |
| rs3820487 | *RGS5* | A | Rec | 1.81 (1.09-3.01) | *0.022834* |
| rs4075958 | *RGS14* | A | Dom | 0.89 (0.73-1.09) | *0.257078* |
| rs4495675 | *RGS18* | C | Rec | 1.19 (0.92-1.53) | *0.181523* |
| rs4501819 | *RGS5* | T | Dom | 1.01 (0.83-1.24) | *0.890237* |
| rs4606 | *RGS2* | G | Add | 1.09 (0.94-1.27) | *0.258192* |
| rs4791223 | *RGS9* | G | Dom | 1.09 (0.87-1.37) | *0.462759* |
| rs511918 | *RGS16* | C | Rec | 0.78 (0.60-1.03) | *0.076287* |
| rs594149 | *RGS16* | G | Dom | 0.84 (0.67-1.04) | *0.109857* |
| rs641875 | *RGS16* | G | Dom | 0.87 (0.72-1.07) | *0.18689* |
| rs6429264 | *RGS7* | A | Dom | 1.20 (0.95-1.51) | *0.125603* |
| rs6473895 | *RGS20* | A | Dom | 0.83 (0.61-1.12) | *0.215129* |
| rs6670735 | *RGS8* | G | Rec | 0.98 (0.69-1.39) | *0.909608* |
| rs6678136 | *RGS4* | A | Rec | 1.36 (1.07-1.73) | *0.012744* |
| rs6689169 | *RGS7* | G | Dom | 0.92 (0.73-1.15) | *0.45369* |
| (continued on following page) | | | | | |

**Supplementary Table 1. All-95 SNPs (Continued)**

| **SNP** | **Gene** | **Minor Allele** | **Model*** | **HR (95%CI)** | ***P*-value** |
| --- | --- | --- | --- | --- | --- |
| rs6693754 | *RGS5* | G | Dom | 1.10 (0.89-1.38) | *0.37702* |
| rs6700378 | *RGS7* | G | Dom | 0.92 (0.73-1.15) | *0.45369* |
| rs694383 | *RGS16* | G | Dom | 0.75 (0.54-1.05) | *0.093632* |
| rs7009781 | *RGS20* | G | Rec | 1.10 (0.75-1.61) | *0.610919* |
| rs7355070 | *RGS5* | G | Dom | 1.15 (0.92-1.44) | *0.220085* |
| rs739999 | *RGS11* | G | Dom | 0.86 (0.67-1.11) | *0.257467* |
| rs7539342 | *RGS5* | A | Dom | 0.88 (0.68-1.13) | *0.326674* |
| rs7540909 | *RGS5* | A | Rec | 0.73 (0.52-1.02) | *0.067476* |
| rs7549021 | *RGS5* | G | Rec | 0.42 (0.22-0.83) | *0.012568* |
| rs762861 | *RGS12* | C | Dom | 1.05 (0.87-1.28) | *0.611996* |
| rs874418 | *RGS12* | A | Dom | 0.88 (0.70-1.09) | *0.24267* |
| rs9427560 | *RGS1* | G | Dom | 1.31 (1.02-1.67) | *0.031854* |
| rs944343 | *RGS3* | C | Add | 0.80 (0.67-0.95) | *0.011796* |

*Model, Rec:Recessive, Dom:Dominant, Add:Additive.
